# Combining Crop Growth Modeling and Statistical Genetic Modeling to Evaluate Phenotyping Strategies

**DOI:** 10.3389/fpls.2019.01491

**Published:** 2019-11-27

**Authors:** Daniela Bustos-Korts, Martin P. Boer, Marcos Malosetti, Scott Chapman, Karine Chenu, Bangyou Zheng, Fred A. van Eeuwijk

**Affiliations:** ^1^Biometris, Wageningen University and Research Centre, Wageningen, Netherlands; ^2^Agriculture and Food, CSIRO, Queensland Bioscience Precinct, QLD, Australia; ^3^School of Agriculture and Food Sciences, The University of Queensland, Gatton, QLD, Australia; ^4^Queensland Alliance for Agriculture and Food Innovation, The University of Queensland, Toowoomba, QLD, Australia

**Keywords:** crop growth model, dynamic traits, wheat, APSIM model, trait hierarchy, genotype to phenotype, P-spline, genomic prediction

## Abstract

Genomic prediction of complex traits, say yield, benefits from including information on correlated component traits. Statistical criteria to decide which yield components to consider in the prediction model include the heritability of the component traits and their genetic correlation with yield. Not all component traits are easy to measure. Therefore, it may be attractive to include proxies to yield components, where these proxies are measured in (high-throughput) phenotyping platforms during the growing season. Using the Agricultural Production Systems Simulator (APSIM)-wheat cropping systems model, we simulated phenotypes for a wheat diversity panel segregating for a set of physiological parameters regulating phenology, biomass partitioning, and the ability to capture environmental resources. The distribution of the additive quantitative trait locus effects regulating the APSIM physiological parameters approximated the same distribution of quantitative trait locus effects on real phenotypic data for yield and heading date. We use the crop growth model APSIM-wheat to simulate phenotypes in three Australian environments with contrasting water deficit patterns. The APSIM output contained the dynamics of biomass and canopy cover, plus yield at the end of the growing season. Each water deficit pattern triggered different adaptive mechanisms and the impact of component traits differed between drought scenarios. We evaluated multiple phenotyping schedules by adding plot and measurement error to the dynamics of biomass and canopy cover. We used these trait dynamics to fit parametric models and P-splines to extract parameters with a larger heritability than the phenotypes at individual time points. We used those parameters in multi-trait prediction models for final yield. The combined use of crop growth models and multi-trait genomic prediction models provides a procedure to assess the efficiency of phenotyping strategies and compare methods to model trait dynamics. It also allows us to quantify the impact of yield components on yield prediction accuracy even in different environment types. In scenarios with mild or no water stress, yield prediction accuracy benefitted from including biomass and green canopy cover parameters. The advantage of the multi-trait model was smaller for the early-drought scenario, due to the reduced correlation between the secondary and the target trait. Therefore, multi-trait genomic prediction models for yield require scenario-specific correlated traits.

## Background

With the availability of low-cost genotyping, genomic prediction has become an attractive tool to increase the number of genotypes considered for selection (Poland et al., 2012; Crossa et al., 2013; Hickey et al., 2014) and to speed up the breeding cycle (Cooper et al., 2014; Haghighattalab et al., 2016; Araus et al., 2018). In genomic prediction, additive and non-additive effects for the target trait (e.g. yield) are estimated in a training set of genotypes, which has genotypic and phenotypic observations. Those estimates are used to predict the phenotypes of the collection of genotypes for which no phenotypic information is available (Meuwissen, 2007).

Complex target traits like yield show low genomic prediction accuracy because they frequently suffer from low heritability and are regulated by a large number of loci with small effects (Crossa et al., 2013; Sorrells, 2015). Yield can be decomposed into a number of underlying genetically correlated traits, called “secondary traits” (Rutkoski et al., 2016; van Eeuwijk et al., 2019) or “components” (Porter and Gawith, 1999; Chapman and Edmeades, 2010). Secondary traits can be either basic traits or intermediate traits. Basic traits correspond to response mechanisms/sensitivities to the environmental conditions (e.g. sensitivity to photoperiod, water uptake capacity, radiation use efficiency). Intermediate traits result from the integration of a number of processes over time (e.g. biomass, flowering time, grain number). As yield and its secondary traits are genetically-correlated, modeling them simultaneously increases yield genomic prediction accuracy, compared to single-trait prediction (Dekkers, 2007; Calus and Veerkamp, 2011; Jia and Jannink, 2012; Alimi, 2016; Biscarini et al., 2017; Sun et al., 2017). In some cases (in small plots, for example), breeders may wish to use the secondary traits directly for selection (e.g. for screening maturity or crop height) within season and discard unwanted genotypes prior to harvest for the next generation of testing. In this case, the interest may be in correlating secondary traits in small plots with expected yield in larger plots in the next season.

Phenotyping additional secondary traits implies an investment that does not always pay off through a larger prediction accuracy for the target trait. Therefore, it is crucial to estimate in advance whether a phenotyping strategy for intermediate traits is likely to increase the prediction accuracy of the target trait. An increased multi-trait prediction accuracy is observed when the heritability of the secondary traits is larger than that of the target trait, and when secondary and target traits are sufficiently genetically correlated (Isik et al., 2017). The evaluation of heritability and genetic correlations is especially relevant for high-throughput phenotyping (HTP). HTP makes the phenotyping of additional traits affordable but it may suffer from larger measurement error than direct (and often destructive) measurements. A large measurement error for secondary traits reduces their heritability and simultaneously reduces the prediction accuracy of the target trait in a multi-trait prediction model (Cabrera-Bosquet et al., 2012; Araus and Cairns, 2014; Yang et al., 2014; Haghighattalab et al., 2016; Rutkoski et al., 2016). The genetic correlation between traits changes over time and across environmental conditions (Crain et al., 2018; Bustos-Korts et al., 2019). Therefore, the potential of secondary traits to improve the prediction accuracy of the target trait is time- and environment-dependent, making it relevant to have a good characterization of the target population of environments (TPE; Chenu, 2015).

A strategy to evaluate the potential of phenotyping strategies is to combine crop growth models and statistical-genetic models to simulate data that resembles the multi-trait data that could be collected in phenotyping experiments. Such simulated data allows to investigate the structure of G × E, and the dynamics of trait correlations and heritability over time. Simulated multi-environment data of traits over time is also useful to evaluate statistical prediction models and test hypotheses regarding crop adaptation (Cooper et al., 2002; Bustos-Korts et al., 2019). Agricultural Production Systems Simulator (APSIM) belongs to a class of widely-used crop growth models, which considers characteristics of the crop, weather, soil, agronomic management, and their interactions over time (Wang et al., 2002; Keating et al., 2003; Holzworth et al., 2014; Chenu et al., 2017). The algorithms in APSIM predict yield as a nonlinear combination of secondary phenotypes, which are calculated indirectly from environmental conditions and from a number of physiological parameters (Wang et al., 2002; Keating et al., 2003; Holzworth et al., 2014). APSIM physiological parameters correspond to basic physiological mechanisms, at the bottom of the trait hierarchy, that modulate crop response to the environmental conditions and can be regarded as constant across environments (Cooper et al., 2002; Hammer et al., 2016; Bustos-Korts et al., 2019). APSIM physiological parameters involve development, capture and use efficiency of environmental resources and biomass partitioning to the different plant organs. Genotypes can differ in their parameter values, leading to phenotypic differences for yield and intermediate traits across environments. Examples of phenotype prediction across environments using APSIM with genotype-dependent parameters can be found in Chapman et al. (2003), Chenu et al. (2009; 2011; 2013), Hammer et al. (2014), and Zheng et al. (2012; 2013). Further discussion about the combination of crop growth models and statistical models can be found in Bustos-Korts et al. (2016b; 2018), van Eeuwijk et al. (2019), and Wang et al. (2019).

Simulated data of secondary and target traits over the growing season present a useful resource to evaluate the advantages of additional phenotyping of traits at different levels of the trait hierarchy and in contrasting environmental conditions (Chapman, 2008). Intermediate traits can be measured at a single time point, or they can be monitored at multiple time points during the season to describe their dynamics. Monitoring traits over time provides useful information about the genotypic response to the environmental conditions integrated over the growing season, providing more insight about the adaptive mechanisms than single traits. Therefore, we might find these dynamics to be more informative about genotypic performance than the collection of single-time point measurements (Malosetti et al., 2006; van Eeuwijk et al., 2010; Hurtado et al., 2012; Hurtado-Lopez et al., 2015). Simultaneous modeling of data points over time is also a strategy to reduce the measurement error and to increase the heritability of traits measured with HTP (Rutkoski et al., 2016).

In this paper, we propose a strategy based on the combination of statistical-genetic and crop growth models to generate data that is similar to those collected in real phenotyping experiments. Such simulated data will be used to evaluate phenotyping strategies. We compare different methods to model trait dynamics over time (i.e. P-splines, nonlinear regression and polynomial models), using an Australian wheat panel simulated with APSIM to grow in a sample of environments representing water deficit patterns present in the Australian TPE. We also discuss and illustrate the convenience of using traits belonging to different levels of the trait hierarchy.

## Methods

### Simulated Data

**Figure 1:**
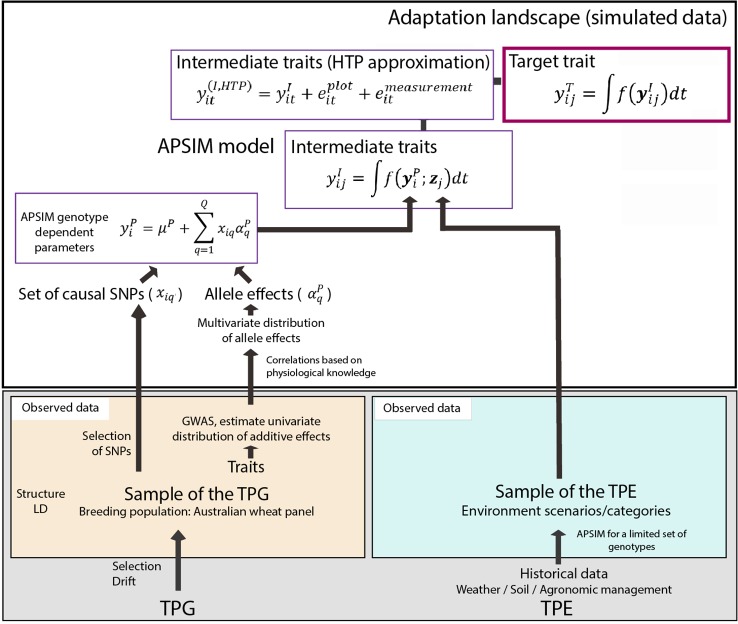
Simulation steps to generate phenotypes for a set of genotypes across environments. Bottom left; an Australian wheat panel is defined as a sample of the target population of genotypes. For this sample of genotypes, phenotypic data for yield and heading date have been collected in eight field trials as well as single nucleotide polymorphisms (SNP) data. The phenotypic data are associated with SNP data in univariate genome-wide association study analyses. From these analyses, empirical distributions for the additive effects of quantitative trait loci underlying these phenotypes are obtained. Physiological knowledge on trait correlations is used to define genetic correlations between Agricultural Production Systems Simulator (APSIM) parameters $\left(y_{i}^{P}\right)$. These correlations are included in a multi-variate description of the quantitative trait loci underlying APSIM parameters. From this distribution, genotype specific APSIM parameters $\left(y_{i}^{P}\right)$ are generated and assigned to a subset of SNPs. Bottom right; we have historical environmental data defining the target population of environments (TPE). We use APSIM to identify environment scenarios (water deficit patterns). The environmental data of the selected scenarios and the genotype-dependent APSIM physiological parameters are used to generate intermediate traits over time $\left(y_{ij}^{I}\right)$. In a breeding programme, these intermediate traits are unknown, but we can approximate intermediate traits by high throughput phenotyping techniques, where the intermediate traits will come with plot $\left(e_{ij}^{plot}\right)$ and measurement error $\left(e_{ij}^{measurement}\right)$. The target trait $\left(y_{ij}^{T}\right)$ is modeled as a function of intermediate traits.

**Figure 2:**
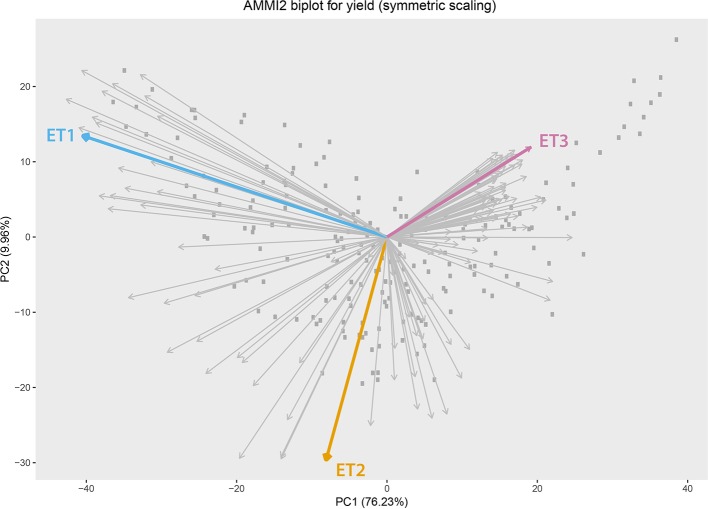
Additive main effect and multiplicative interaction biplot for grain yield in Emerald, Merredin, Narrabri, and Yanco during 1993–2013. Gray squares represent genotype scores and grey arrows represent environment scores. Environments that were sampled from different environment types (ET) for a more detailed characterization of traits over time are indicated in coloured arrows. ET1 represents trials without water deficit (represented in the sample by “Yanco_2010”), ET2 corresponded to intermediate drought starting around flowering (represented by “Narrabri_2008”). ET3 corresponded to intense drought starting early during the growing season (represented in the sample by “Emerald_1993”).

Simulated data consisted of yield, daily biomass and green canopy cover, for 199 genotypes in three Australian environments with contrasting water deficit patterns (**Figure 1**). These three environments were sampled from a total of 124 environments (4 locations and 31 seasons) corresponding to the TPE. These three environments were chosen to represent target drought environment types (ETs) that are relevant to Australian wheat production. ET1 have no or short-term water limitation and was represented in the sample by “Yanco_2010.” ET2 corresponded to intermediate drought starting around flowering, represented by “Narrabri_2008.” ET3 corresponded to intense drought starting early during the growing season (around 200^o^Cd before flowering) and was represented by “Emerald_1993,” **Figure 2** (more details in Chenu et al., 2013; Bustos-Korts et al., 2019). Trait correlations changed over time and across environments, building up G × E for grain yield during the growing season. Different traits are expected to confer adaptation to each environment type, making them interesting to study the convenience of phenotyping additional traits to improve yield prediction accuracy.

We simulated phenotypic data in the following steps (**Figure 1**): 1) We generated genotype-specific values for 12 APSIM physiological parameters, regulating phenology, capture of environmental resources, resource use efficiency and biomass partitioning (**Table 2** in Bustos-Korts et al., 2019). These APSIM physiological parameters were regulated by 300 single nucleotide polymorphisms (SNPs) with simulated additive effects sampled from a gamma distribution that followed the same shape and rate as the quantitative trait locus effects for real phenotypic data (see **Figures 1** and **2** in Bustos-Korts et al., 2019). 2) We ran APSIM simulations for the three sampled environments from the TPE. We saved phenology, yield at harvest and the daily output for biomass.

### Plot and Measurement Errors

As APSIM is fully deterministic, the simulated data do not include stochastic fluctuations due to experimental and measurement error. We added some error structures to APSIM output to investigate questions related to phenotyping schedules and multi-trait prediction. Field traits measured with HTP can contain two main sources of error: a plot error due to the within-trial heterogeneity (e.g. plot to plot variation) and a measurement error. This measurement error adds imprecision to the phenotype observed directly (e.g. by harvesting, processing and weighing the biomass). We added an experimental (plot) error and a measurement error to the APSIM output of daily biomass and daily green canopy cover from 20 days after sowing until harvest. Part of this methodology is also described in Bustos-Korts et al. (2017).

#### Size of the Experimental (Plot) and Measurement Errors

To simulate the experimental (plot) error, we considered a heritability of 0.50 for yield and 0.90 for biomass and canopy cover. The plot error size was calculated from Equation (1):

(1) $$H^{2}=\frac{\sigma_{g}^{2}}{\sigma_{g}^{2}+\sigma_{plot}^{2}}$$

In Equation (1), the genotypic variance $\left(\sigma_{g}^{2}\right)$ was assumed to be equivalent to the variance of APSIM biomass for a given day. As APSIM yield and biomass genotypic values do not contain error, phenotypic differences in the same environment can be considered as genetic. The experimental error $\left(\sigma_{plot}^{2}\right)$ was sampled jointly for yield, biomass and green canopy cover from a multivariate normal distribution with a covariance of 0.1 and a variance of 1.0. The covariance between plot error structures for yield and biomass was larger than zero because traits measured on the same plot might be correlated. The phenotypic value for genotype *i* and day *j* was calculated with Equation (2):

(2) $$y_{it}^{\left(I,direct\right)}=y_{it}^{I}+e_{it}^{plot}$$

Where $y_{it}^{I}$ is the APSIM phenotype for an intermediate trait *I*, genotype *i* and day *t* and $e_{it}^{plot}$ is the experimental (plot) error for genotype *i* and day *t*. As the genotypic variance of biomass (or green canopy cover) changes over time, we rescaled the plot error $\left(e_{it}^{plot}\right)$ to keep heritability constant and equal to 0.9 during the growing season.

Besides the experimental (plot) error, we added a measurement error that simulates the HTP approximation of $y_{it}^{direct}$;

(3) $$y_{it}^{\left(I,HTP\right)}=y_{it}^{\left(I,direct\right)}+e_{it}^{m}$$

In Equation (3), $y_{it}^{\left(I,HTP\right)}$ is the phenotype “measured” by HTP, $y_{it}^{\left(I,direct\right)}$ is the phenotypic value for genotype *i* and day *j* and $e_{it}^{m}$ is the simulated measurement error for genotype *i* and day *t* (**Figure 1**). Measurement error $\left(e_{it}^{m}\right)$ was sampled independently for each environment, trait and day (random error). We evaluated two classes of measurement error size; a homogeneous over time and a measurement error size that was a function of canopy cover (details about the measurement error size are given in the following sections).

#### Homogeneous Measurement Error Over Time

The homogeneous measurement error, $e_{it}^{m}$, was considered as constant over time and across genotypes. We examined eight levels of measurement error size $\left(e_{it}^{m}\right)$ on yield prediction accuracy. The size of $e_{it}^{m}$ was defined to achieve an R^2^ between the $y_{it}^{\left(I,HTP\right)}$ and $y_{it}^{\left(I,direct\right)}$ of 0.10, 0.20,…, 0.90. For each of these measurement error levels, the relative size of the measurement error with respect to the phenotypic variance (i.e. the variance of $y_{it}^{\left(I,direct\right)}$) was kept constant over time.

#### Measurement Error as a Function of Canopy Growth

**Figure 3:**
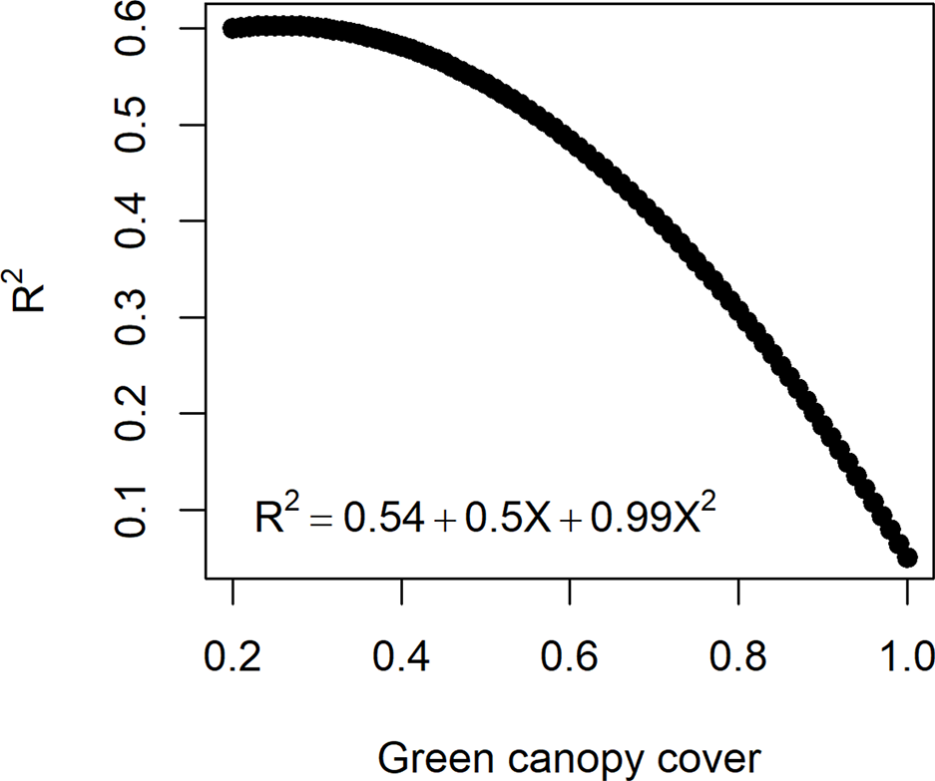
Quadratic function to relate the measurement error size (R^2^ between the high-throughput phenotyping and direct measurement of biomass) to the green canopy cover observed for a genotype at a specific day. As genotypes differ in green canopy cover in a given environment and day, their measurement error is also genotype-specific.

In reality, measurement error size in HTP (estimated by $e_{it}^{m}$) can change over time, depending on the dynamics of other traits, e.g. the error increases as canopy closes and decreases with the onset of senescence (Grieder et al., 2015; Christopher et al., 2016; Magney et al., 2016). The influence of trait dynamics on measurement error can be taken into account when simulating measurement error for biomass. Hence, R^2^ between $y_{it}^{HTP}$ and $y_{it}^{direct}$ was assumed to decrease with a quadratic function with an increase in canopy cover (**Figure 3**). The function that relates the measurement error size to canopy cover was defined in such a way that the maximum R^2^ (smallest measurement error) was 0.6 to agree with experiments reported in the literature (e.g. Grieder et al., 2015; Magney et al., 2016) and a R^2^= 0.1 when the canopy is fully closed. Hence, for a given genotype, the simulated measurement error increases when the green canopy cover increases (**Figure 3**). As the dynamics of canopy cover are genotype dependent, in this measurement error class, the size of the measurement error becomes time- and genotype-dependent.

#### Phenotyping Schedules

Phenotyping schedules were defined by combining the measurement error sizes (9 measurement error sizes for the homogeneous measurement error over time, plus one measurement error as a function of canopy growth) with five levels of measurement intervals (every 5, 10, 15, 20, and 25 days) in a factorial way. Thus, for each environment, we obtained 50 phenotyping schedules differing in their measurement error size and interval between two consecutive measurements.

#### Heritability of APSIM Physiological Parameters

Besides the simulated intermediate traits biomass and canopy cover, we evaluated the impact of using basic traits that are lower in the trait hierarchy, and that correspond to the physiological mechanisms of response to the environment (APSIM physiological parameters) on yield prediction accuracy. We focused on three APSIM physiological parameters that have an important effect on yield across environments, as identified by a factorial regression model applied to 124 environments in Bustos-Korts et al. (2019). These simulated APSIM physiological parameters were radiation use efficiency (“y_rue”), sensitivity to photoperiod (“photop_sens”), and vernalization requirements (“vern_sens”). For each of them, we evaluated a range of H^2^s (from 0.20 to 0.80). The three APSIM physiological parameters with simulated error were included simultaneously in a multi-trait genomic prediction model.

### Statistical Modeling of Phenotypes Over Time

The simulated data, with different error sizes and intervals between measurements, were used to extract parameters of the dynamics for biomass and green canopy cover. These parameters were introduced in multi-trait genomic prediction models to compare prediction accuracy calculated from a single-trait (yield) or from multiple traits modeled simultaneously (biomass, green canopy cover dynamics and yield, or APSIM parameters and yield). In this section, we describe the statistical models used to characterize the dynamics of biomass and green canopy cover during the growing season.

#### Logistic Regression Fitted to Biomass

A logistic function was fitted independently to the simulated biomass HTP data over time for each genotype and phenotyping schedule.

(4) $$y_{t}=\frac{L}{1+e^{-k\left(t-t_{0}\right)}}+\epsilon_{t}$$

In model (4), *y* *_t_* is the simulated biomass (with plot and measurement error) at day *t*, *L* is the curve's maximum value (asymptote), *k* is the initial relative growth rate and *t* *_o_* is the day at which biomass achieves the maximum growth rate (inflection point) and ε*_t_* is a residual. By definition, maximum growth rate in a logistic curve is $\frac{1}{4}kL$. The curve was fitted with the *nls* function of the *stats* package in R (R Core Team, 2016). The estimated parameters will be represented as follows; BL_asy is the asymptote for biomass fitted with a logistic curve, BL_inf is the inflection day for biomass fitted with a logistic curve, BL_slope is the maximum slope of biomass fitted with a logistic curve.

#### Cubic Function Fitted to Green Canopy Cover

A cubic function was fitted independently to the simulated green canopy cover HTP data of each genotype and phenotyping schedule over time, using the *lm* function in R.

(5) $$y_{t}=\beta_{0}+\beta_{1}t+\beta_{2}t^{2}+\beta_{3}t^{3}+\epsilon_{t}$$

The fitted values of Equation (5), were used to calculate the maximum cover (CS_max, defined as the maximum fitted value) and the integral of the fitted curve (CS_int, defined as the sum of the daily fitted values).

#### P-Splines Fitted to Biomass and Canopy Cover

P-splines were fitted to the time series data for biomass during the growing season, using cubical B-splines and second order difference penalties (Eilers and Marx, 1996; Eilers et al., 2015). For the B-splines basis 100 equidistant knots were used. The P-splines were fitted as a mixed model (Currie and Durban, 2002)

(6) $$y_{t}=\beta_{0}+\beta_{1}t+\underset{k}{\sum }b_{k}v_{k}\left(t\right)+\epsilon_{t}\;$$

In Equation (6), *y* *_t_* is biomass at time point *t*, *β* *_0_* is the intercept, *β* *_1_* is the slope for the linear trend over time $\underset{k}{\sum }b_{k}v_{k}\left(t\right)$, is the non-linear trend over time, and *є* *_t_* is the residual. The original B-splines basis functions were transformed to the non-linear trend functions *v* *_k_* *(t)* using spectral decomposition of the penalty matrix (Wand and Ormerod, 2008). To summarize the curves of simulated biomass and green canopy cover over time, we calculated the following parameters; BS_asy, which is the asymptote for biomass calculated from a spline fit, calculated as the biomass fitted values at the last day of the growing season, BS_inf, which is the inflection day for biomass, calculated as the day in which the maximum of the spline first derivative occurs, BS_slope, which is the maximum slope for biomass calculated from the first derivative of the B-spline basis. A description of the curve parameters is also given in **Table 1**.

**Table 1:** Abbreviations used to describe the parameters estimated for the parametric and the P-spline models fitted to biomass and canopy cover over time.

Abbreviation	Description
BL_asy	Asymptote for biomass fitted with a logistic curve
BS_asy	Is the asymptote for biomass calculated from a spline fit
BL_inf	Inflection day for biomass fitted with a logistic curve
BL_slope	Maximum slope of biomass fitted with a logistic curve
BS_inf	Inflection day for biomass calculated from a spline fit
BS_slope	Maximum slope for biomass calculated from a spline fit
CC_int	Integral for green canopy cover calculated from the fit of a cubic curve
CC_max	Maximum green canopy cover calculated from the fit of a cubic curve
CS_int	Integral for green canopy cover calculated from a spline fit
CS_max	Maximum green canopy cover calculated from a spline fit

#### Heritability of Curve Parameters

To estimate the repeatability (“heritability”), we fitted the parametric models and the splines twice; first to the data with plot and measurement error, and then to the data without error (the APSIM output). We estimated the curve parameters for both data sets. To get an approximation of the heritability, we calculated the R^2^ between the curve parameters extracted from the logistic, cubic polynomial or spline fitted to the data with error, and those observed for the APSIM output without error.

### Genomic Prediction

#### Single Trait Predictions (Yield)

Single trait genomic prediction for yield was carried out with the Genomic Best Linear Unbiased Prediction model.

(7) $$y_{i}=\mu +G_{i}+\epsilon_{i}$$

In Equation (7), *y* *_i_* is yield of genotype *i*, *µ* is the intercept, *G* *_t_* stands for the random genotype effects that follow $G_{i}\sim MVN\left(0,\Sigma \right)$ where *∑* is a covariance matrix. The variance-covariance matrix *∑* is modeled as *∑*=*∑* *_G_*, where *∑* *_G_* is the genotypic kinship matrix, calculated as in Astle and Balding (2009). The predictions were made with ASReml 3.0 (VSN-International, 2015).

#### Multi-Trait Predictions

Multi-trait genomic prediction models fitted on i) APSIM yield output and parameters extracted from the dynamics of simulated biomass and canopy cover, or ii) APSIM yield and APSIM physiological parameters with error.

(7) $$y_{ik}=\mu +T_{k}+G_{ik}+\epsilon_{ik}$$

In model (8), *y* *_ik_* is the phenotype for genotype *i* and trait *k*, *µ* is the intercept, *T* *_k_* is the fixed main effect of trait *k*. *G* *_ik_* is the random effect for genotype *i* and trait *k*, following *G* *_ik_* ∼ *MVN (0,∑* ^*^ *)*. The variance–covariance matrix *∑* ^*^ is modeled as *∑*=*∑* *_G_*⨂*∑* *_T_*, where *∑* *_G_* is the same genotypic kinship matrix that was used in Equation (7). *∑* *_T_* is the variance-covariance between traits, modeled with an unstructured model and ⨂ is the Kronecker product. *є* *_ik_* ∼ *MVN (0,∑* ^*^ *)*, where *R* is a diagonal matrix, allowing for trait-specific residuals.

Several combinations of traits were evaluated in model (8), starting with a full model with all the spline or parametric curve parameters for both biomass and canopy cover over time. Traits that did not contribute to increase yield prediction accuracy were removed from the model. The final multi-trait model considered the following traits; biomass asymptote (BL_asy and BS_asy), the maximum slope of biomass accumulation (BL_slope, BS_slope) and maximum canopy cover (CL_max, CS_max). For the APSIM physiological parameters, we included radiation use efficiency (“y_rue”), sensitivity to photoperiod (“photop_sens”), and vernalization requirements (“vern_sens”). These APSIM physiological parameters were selected because they were important for G×E in this data sets, based on previous analyses (Bustos-Korts et al., 2019). A detailed list of the single- and multi-trait prediction models is given in **Table 2**.

**Table 2:** Single and multi-trait genomic prediction models. Details about the trait description are indicated in **Table 1**.

Model	Traits	Phenotyping error
M1	Yield	Only plot error (H^2^= 0.5)
M2S	Yield, BS_asy, BS_slope, CS_max	Sampled independently for each day, with a constant variance. Evaluated a range of H^2^= 0.1 to H^2^= 0.9
M2P	Yield, BL_asy, BL_slope, CC_max	Sampled independently for each day, with a constant variance. Evaluated a range of H^2^= 0.1 to H^2^= 0.9
M3S	Yield, BS_asy, BS_slope, CS_max	A quadratic function of genotype-specific canopy cover
M3P	Yield, BL_asy, BL_slope, CC_max	A quadratic function of genotype-specific canopy cover
M4	Yield, y_rue, photo_sens, vern_sens	Independently sampled for each trait, evaluating a range of H^2^= 0.2 to H^2^= 0.8

#### Prediction Scenarios

All the multi-trait genomic prediction models (**Table 2**) were evaluated in two prediction scenarios; *nG_all* and *nG_yield*. In *nG_all*, all traits (i.e. yield and secondary traits) were present in the training set, but they were missing in the validation set. In *nG_yield*, secondary traits were present in the training and validation set, and only yield was missing in the validation set.

#### Prediction Accuracy

Prediction accuracy was calculated as the Pearson correlation coefficient between APSIM yield (genotypic value) and the predicted phenotypes (Meuwissen et al., 2001), considering a training set of 100 genotypes and a validation set of 99 genotypes. Thirty training sets were constructed with the uniform sampling method described by Bustos-Korts et al. (2016a)and by Jansen and van Hintum (2007). To comply with the normality assumption, correlation means and standard errors across 30 training set realizations were calculated on a transformed scale using Fischer's z transformation, $z=\frac{1}{2}\;\left(\ln\left(\frac{1+r}{1-r}\right)\right).$. Then, means and the confidence interval lower and upper bound were back transformed using $r=\frac{\exp\left(2z\right)+1}{\exp\left(2z\right)-1}$ before reporting them.

## Results

We used the APSIM-simulated traits to investigate the structure and the magnitude of G × E, trait correlations over time and across environments and to evaluate multi-trait prediction models. We would like to emphasize that when we mention traits like “yield,” “biomass,” or “canopy cover,” we refer to simulated traits.

### Patterns of Trait Correlations Over Time Depend on the Environment

**Figure 4:**
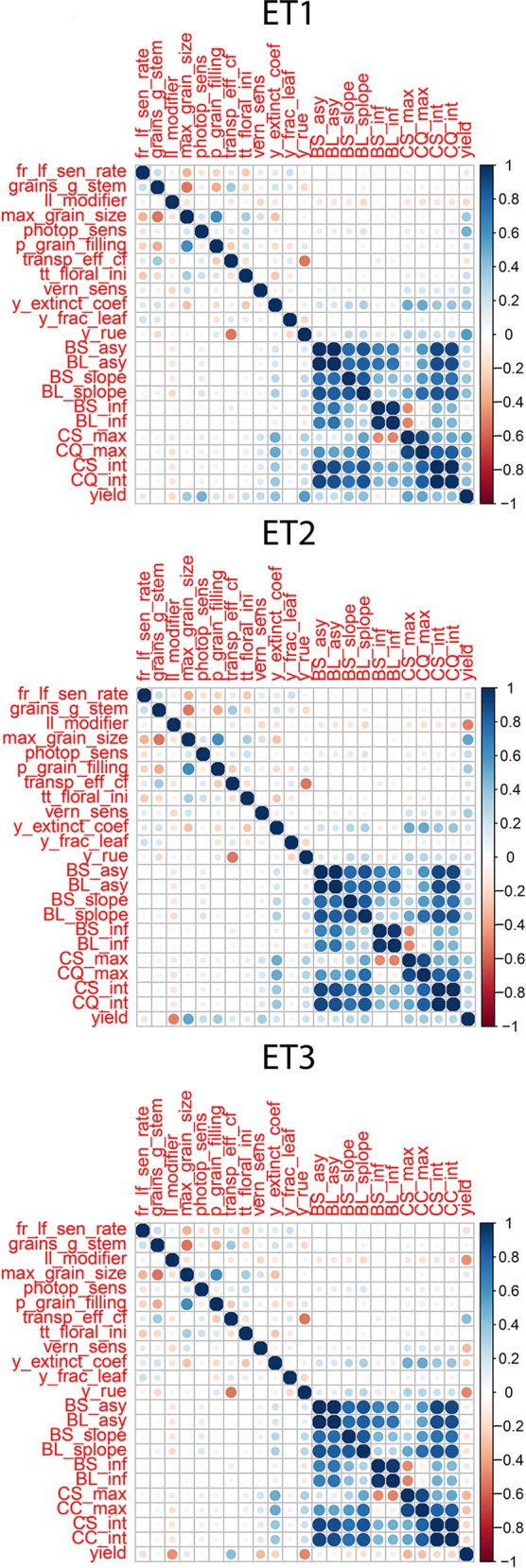
Correlations between APSIM parameters, parameters of biomass accumulation and canopy cover and yield. Details about the trait description are indicated in **Table 1**.

We observed the AMMI biplot shown in Bustos-Korts et al. (2019) to select three environments that represent target production ETs for the Australian wheat belt (**Figure 2**). These ETs have a large G × E that is driven by water deficit patterns; ET1 has no water limitation, ET2 has mild drought starting around flowering, and ET3 has intense drought, starting early during the growing season. Correlations between traits were largely affected by the environmental conditions, with a strong correlation between yield and biomass in environments without water limitation (ET1), and with a moderate correlation between them in dry environments like ET3 (**Figure 4**). Trait correlations also changed during the growing season, depending on the progression of the water stress and the environmental conditions over time (**Figure 7** in Bustos-Korts et al., 2019); i.e. the correlation between biomass and final yield was intermediate in the late-stress environment ET2 and large (>0.80) in the non-stress environment and ET1, whereas in the dry environment ET3, the correlation was negative at the beginning of the growing season and became positive after heading. The temporal changes in trait correlations give insight about which traits are contributing to end-of-season yield outcomes at specific moments within the season. These dynamics also influence the potential of secondary traits like biomass or canopy cover to improve prediction accuracy of the target trait when included simultaneously in a multi-trait model (**Figure 8** in Bustos-Korts et al., 2019). Conversely, trait correlations are also a diagnostic tool about the environmental conditions experienced by the crop, and can therefore be used to classify environments.

**Figure 5:**
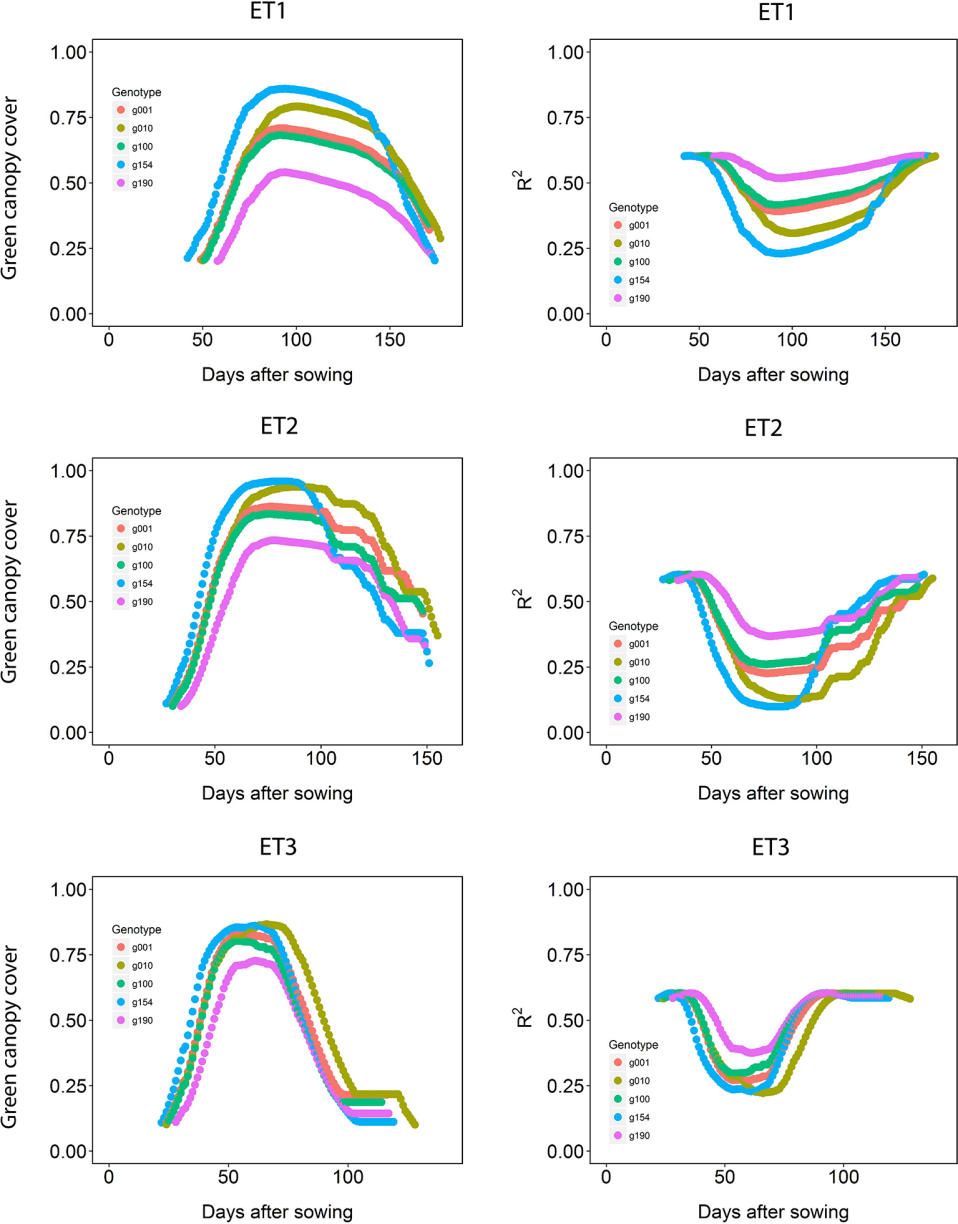
Green canopy cover dynamics for a random sample of five genotypes (left panels) and genotype-specific R^2^ between high-throughput phenotyping and direct measurement of green canopy cover during the growing season (right panels) in three trials representing different environment types (i.e. different patterns of drought).

The dynamics of canopy cover also depended on the genotype and on the environmental conditions (**Figure 5**, left panels). Senescence began earlier in ET3, due to more rapid development associated with higher growth temperatures (**Table 1** in Bustos-Korts et al., 2019). The genotypic differences in the dynamics of green canopy cover influence the rate size of the measurement error (**Figure 5**, right panels); dry environments like ET3 have a faster increase in canopy cover in the early season, and an earlier reduction in canopy cover, and therefore they have relatively a greater proportion of the season with a smaller measurement error (larger R^2^).

### Correlations Between Parameters for Secondary Traits and the Target Trait Depend on the Environment Type

Multi-trait prediction accuracy is influenced by the H^2^ and the correlation between secondary and target traits. As expected, the correlations between yield and the parameters of the secondary traits, biomass and green canopy cover followed the same trends as the biomass and green canopy cover without error; BS_asy and BL_asy were positively correlated to yield in ET1 and ET2 (stronger in ET1 than in ET2), and negatively correlated to yield in ET3 (**Figure 4**). This implies that breeders need to change their selection strategy, considering the traits that contribute to adaptation in each environment type. BS_slope and BL_slope were also correlated to yield (**Figure 4**), following the same environment-dependent correlation sign as BS_asy and BL_asy (positive in ET1 and ET2, and negative in ET3. However, the correlations were a stronger for BS_slope and BL_slope than BS_asy and BL_asy. BS_inf and BL_inf were only correlated to yield in ET3, probably related to the more asymmetric and irregular biomass accumulation dynamics in ET2 and ET1. As for biomass accumulation, maximum green canopy cover (CS_max and CL_max) were also positively correlated in ET1 and ET2, and they were negatively correlated with yield in the dry environment ET3. This pattern in the trait correlations supports the idea that, in dry environments with little in-season rain, smaller canopies allow for more effective use of water throughout the growing season, avoiding the early depletion of soil water. In those same environments, a fast-growing genotype can use too much water and be stressed around flowering at the critical time when grains are being set (and maximum yield in that season becomes fixed, and is realised by water supply during grain-filling). This pattern also indicates that different traits need to be phenotyped for multi-environment prediction, depending on the environment-type and that the selection pressure applied by breeders on specific traits needs to be adjusted for each environment type. Therefore, it is essential to have an adequate environment characterization before deciding which traits to include in the phenotyping schedule.

### Modeling Phenotype Dynamics as Measured by HTP Increases Heritability

We simulated HTP measurements for biomass and green canopy cover by adding a plot and a measurement error to the daily APSIM output for both of these traits. We used the simulated data to evaluate a number of configurations for measurement error size and phenotyping interval (expressed as the number of days between two consecutive measurements). We considered two scenarios for measurement error size; a constant error size over time (with nine levels), and a measurement error size that changes over time as a function of green canopy cover. The simulated HTP data for biomass and green canopy cover were fitted with parametric models (logistic or cubic function), and P-splines. The parameters extracted from biomass and green canopy cover over time were used to evaluate how HTP schedules influence prediction accuracy for the target trait. In this section, we describe the H^2^ for parameters of the logistic curve and for parameters defined on the basis of the fitted P-spline function, as a rough indicator for the potential of that parameter to predict yield.

**Figure 6:**
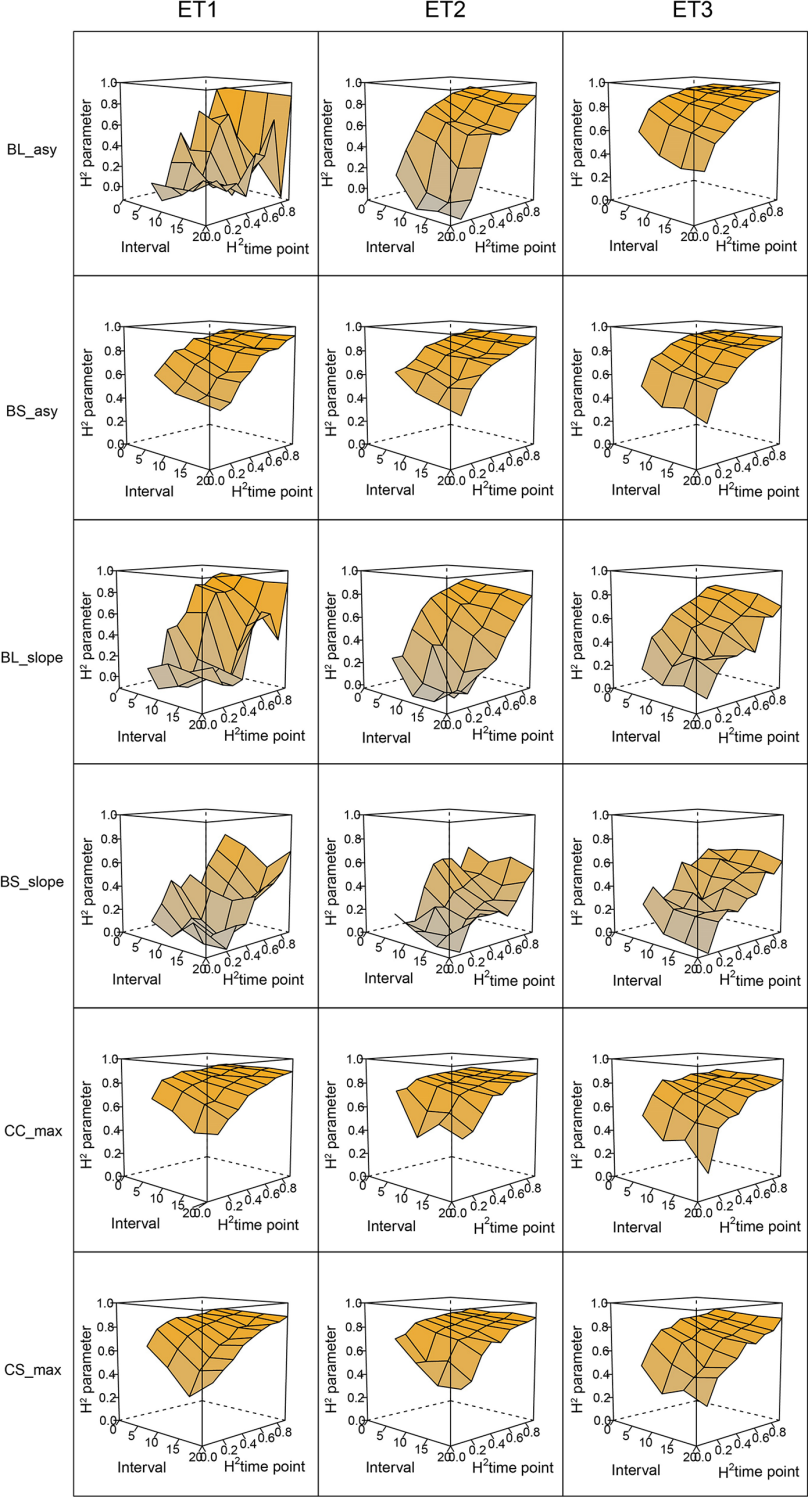
Heritability of the parameters from curves fitted to the dynamics of biomass accumulation and green canopy cover for the collection of genotypes, measured with high-throughput phenotyping (HTP). BL_asy is the asymptote for biomass fitted with a logistic curve, BS_asy is the asymptote for biomass fitted with a spline, BL_slope is the maximum slope of biomass fitted with a logistic curve, BS_slope is the maximum slope of biomass fitted with a spline, CC_max is maximum green canopy cover calculated from a cubic curve and CS_max is maximum green canopy cover calculated from a spline fit. The x-axis indicates the interval for different analyses, expressed as the number of days (5, 10, 15, or 20) between two consecutive HTP measurements. The z-axis (H^2^ time point) indicates the quality of the HTP measurement, quantified as the R^2^ between the direct phenotypic measurements (APSIM biomass plus plot error, Equation 2) and HTP (APSIM biomass plus plot and measurement error, Equation 3).

**Figure 7:**
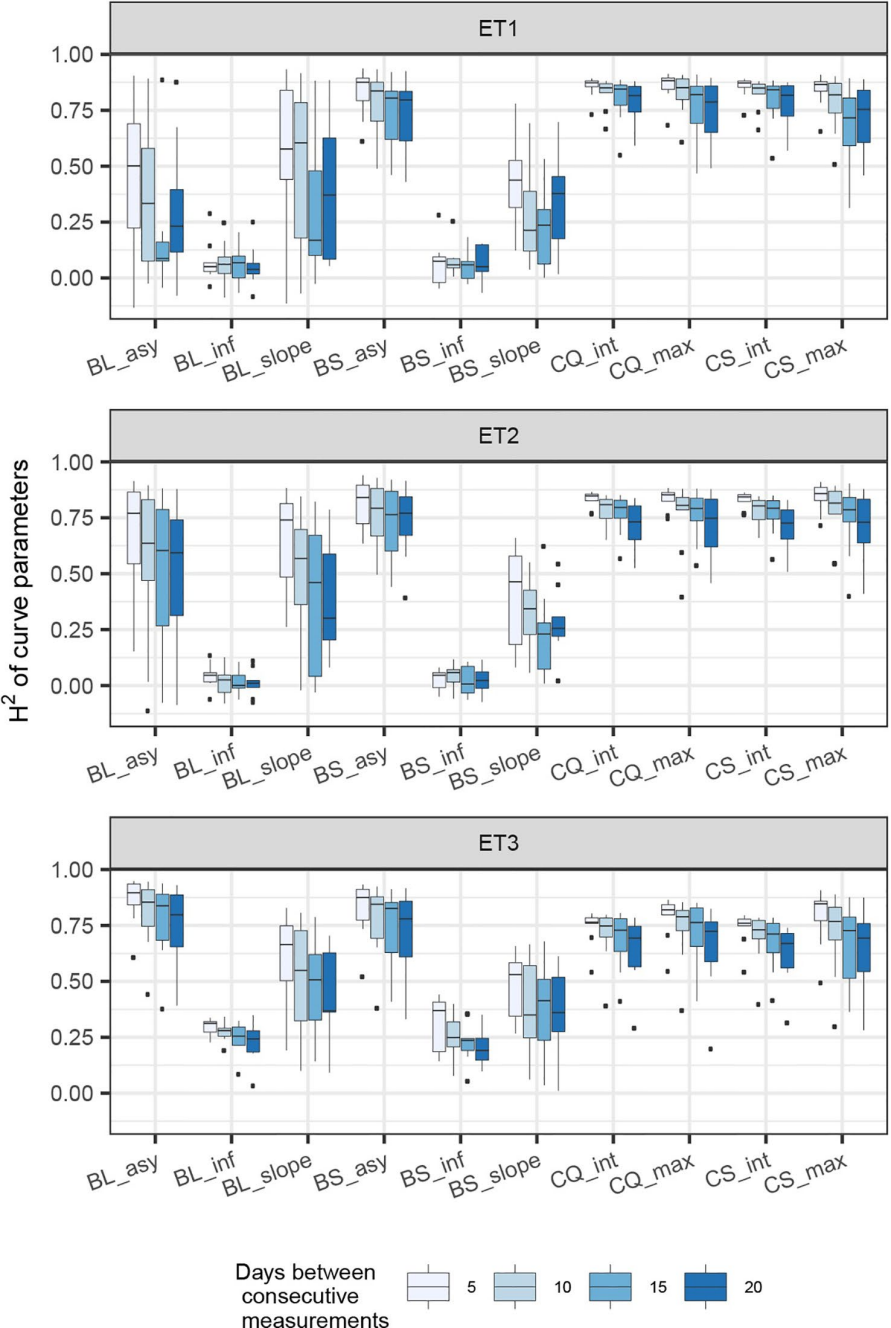
Heritability of curve estimates, as a function of the heritability at single time points. Each box contains H^2^ estimates obtained across levels for interval size between two consecutive measurements. Details about the trait description are indicated in **Table 1**.

**Figure 8:**
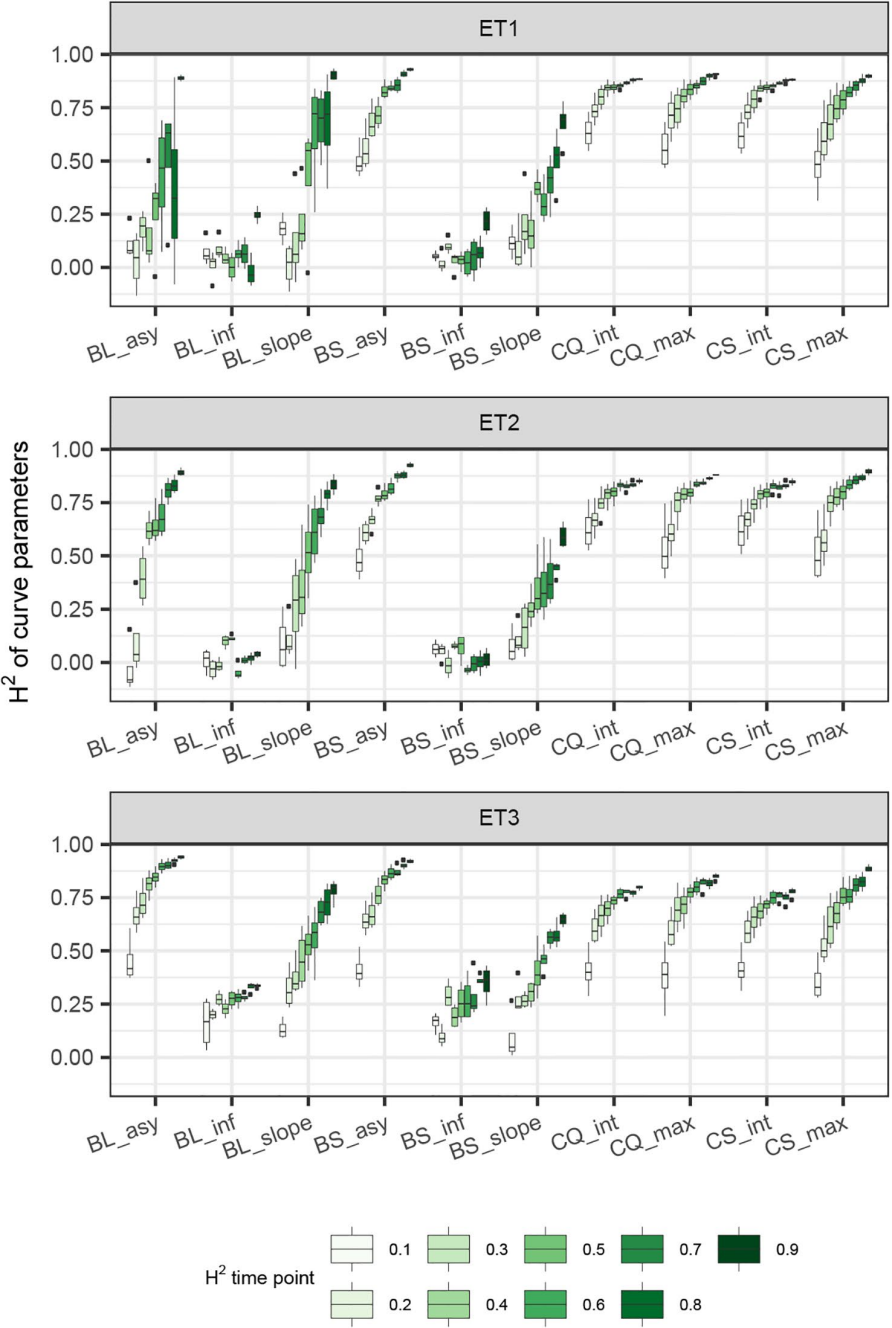
Heritability of curve estimates, as a function of the interval between two consecutive measurements, expressed in days. Each box contains H^2^ estimates obtained across measurement error sizes. Details about the trait description are indicated in **Table 1**.

In general, the H^2^s of parameters for biomass and green canopy cover over time were substantially larger when using P-splines, than when using parametric models (**Figures 6**–**8**). The logistic model led to a more variable response of H^2^ in relation to the measurement interval and to the H^2^ of individual measurements. This was because of the lack of fit of the logistic curve when there were few measurements (intervals of 20 days). The H^2^ for the parameters of the logistic curve fitted to HTP biomass data over time was largest in ET2 and ET3, where biomass curves were more symmetric. In ET1, biomass accumulation over time was most asymmetric (**Figure 6** in Bustos-Korts et al. 2019) and H^2^ for the parameters of the logistic curve was, therefore, lower in this environment. In the three environments, H^2^ increased with more frequent (smaller interval between two consecutive measurements) and with more precise measurements at individual time points (larger R^2^ between the direct phenotypic measurements, Equation 2, and HTP, Equation 3).

When comparing the H^2^ of the three parameters of the logistic curve fitted to biomass accumulation over time, we observed that the asymptote and the inflection point showed a somehow flat H^2^ surface (**Figure 6**), indicating that precise estimates of these parameters can be obtained, even after reducing measurement frequency and increasing the measurement error. For example, in ET3, the same H^2^ estimate for the asymptote can be obtained (H^2^∼0.80) from an HTP technology that delivers an R^2^ between HTP and direct measurements of 0.50 or with one that has an R^2^ of 0.80. The same applies for measurement intervals; if multiple time points are measured simultaneously, the same mean H^2^ can be obtained measuring every 5 or every 15 days. This highlights the convenience of integrating measurements over time, compared to using single time points independently.

We also used P-splines to extract parameters for the dynamics of biomass and green canopy cover over time. For the P-splines, similar H^2^ was obtained for curve parameters across environments (**Figures 6**–**8**), showing that P-splines are a more flexible model than the logistic curve. Therefore, P-splines can accommodate the asymmetries of the biomass accumulation curve. The H^2^ achieved for the spline fitted values was also larger than the H^2^ of the logistic curve and the H^2^ surface was more smooth (**Figures 6**–**8**). The smoother H^2^ surface and the reduced variation indicate that P-splines are better than the logistic curve when it comes to removing part of the measurement error by integrating information throughout the season. In practice, this means that, when using a spline to integrate the HTP measurements for biomass, measurements can be done at a lower frequency (larger intervals) and lower precision (lower R^2^ between HTP and APSIM biomass) to still obtain large H^2^, compared to the logistic model. We characterized the P-splines as fitted to the HTP measurements for biomass by the following parameters; asymptote (BS_asy), maximum biomass accumulation rate (BS_slope) and the inflection point of biomass accumulation (BS_inf). The largest H^2^ was obtained for BS_asy. The H^2^ of BS_slope was slightly lower (H^2^∼0.60–0.70) and the lowest H^2^ was observed for BS_inf (H^2^∼0.10–0.30, **Figures 7** and **8**). This implies that BS_slope was more difficult to estimate, requiring very frequent and precise measurements to obtain a large H^2^.

### Multi-Trait Predictions Using Secondary Traits

**Figure 9:**
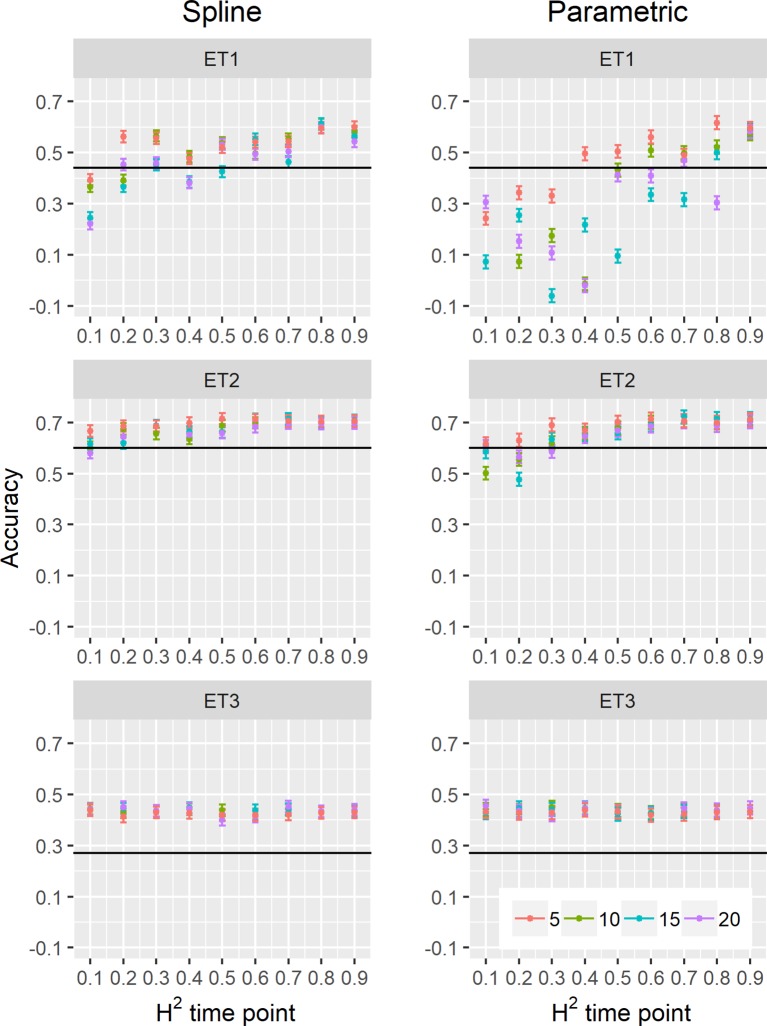
Yield prediction accuracy and standard error in ET1, ET2, and ET3 calculated with the multi-trait prediction models M2S and M2P, considering yield and parameters estimated from the biomass and green canopy cover dynamics using P-splines (BS_asy, BS_slope and CS_max) or parametric models (BL_asy, BL_slope, or CL_max), for the scenario *nG_allt* (target and secondary traits missing in the validation set). The x-axis indicates the heritability of individual time points measured with HTP, quantified as the R^2^ between the direct phenotypic measurements (APSIM biomass plus plot error, Equation 2) and HTP (APSIM biomass plus plot and measurement error, Equation 3). Symbol colour indicates the interval, expressed as the number of days between two consecutive HTP measurements. Black horizontal lines shows yield prediction accuracy for a single trait model trained with yield data for the genotypes in the training set (M1). Single- and multi-trait models were trained with 100 genotypes, whereas 99 genotypes were used for validation. Bars indicate the confidence interval for the mean, calculated across 30 realizations of the training-validation sets.

**Figure 10:**
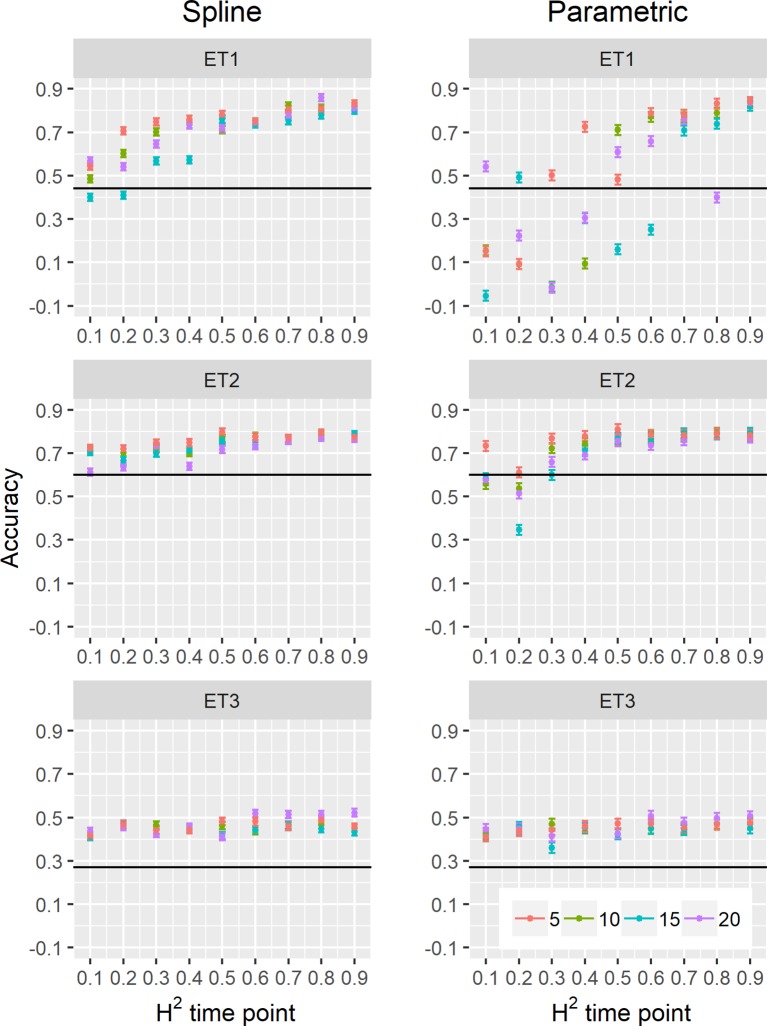
Yield prediction accuracy and standard error in ET1, ET2, and ET3 calculated with the multi-trait prediction models M2S and M2P, considering yield and parameters estimated from the biomass and green canopy cover dynamics using P-splines (BS_asy, BS_slope, and CS_max) or parametric models (BL_asy, BL_slope, or CL_max), for the scenario *nG_yld* (target trait missing in the validation set, but secondary traits are present in both training and validation set).The x-axis indicates the heritability of individual time points measured with HTP, quantified as the R^2^ between the direct phenotypic measurements (APSIM biomass plus plot error, Equation 2) and HTP (APSIM biomass plus plot and measurement error, Equation 3). Symbol colour indicates the interval, expressed as the number of days between two consecutive HTP measurements. Black horizontal lines shows yield prediction accuracy for a single trait model trained with yield data for the genotypes in the training set (M1). Single- and multi-trait models were trained with 100 genotypes, whereas 99 genotypes were used for validation. Bars indicate the confidence interval for the mean, calculated across 30 realizations of the training-validation sets.

We estimated parameters of logistic, cubic curves or spline fitted values for biomass accumulation and green canopy cover during the growing season with HTP measurement error. We used those parameters as correlated traits for yield genomic prediction. In general, multi-trait genomic prediction models (**Figure 9**) had a larger accuracy than single-trait models (**Figures 6**–**9**). However, the prediction accuracy of multi-trait models was highly dependent on the quality (H^2^) of the correlated trait and on the correlation between the secondary traits and the target trait; more frequent and more precise HTP measurements led to larger accuracy, compared to less frequent and less precise measurements, only if traits were correlated. Therefore, prediction accuracy had a very large increase in ET1 (from 0.27 to 0.60) whereas it showed a moderate increase in ET2 (from 0.60 to 0.73) and it did not increase in ET3. The increase in prediction accuracy was more consistent (less variation between phenotyping schedules) when using P-splines than when using the parametric models (**Figures 9** and **10**). This is related to the smaller variation in the estimates of curve summaries when using P-splines, than when using the parametric models (**Figures 6**–**8**). When comparing different phenotyping schedules, we observed that the differences in prediction accuracy between the different measurement intervals become more evident when the H^2^ of individual measurements is low. In other words, if H^2^ of individual time points is small, multi-trait prediction accuracy benefits from more frequent measurements and from modeling secondary traits over time (**Figures 9** and **10**).

**Figure 11:**
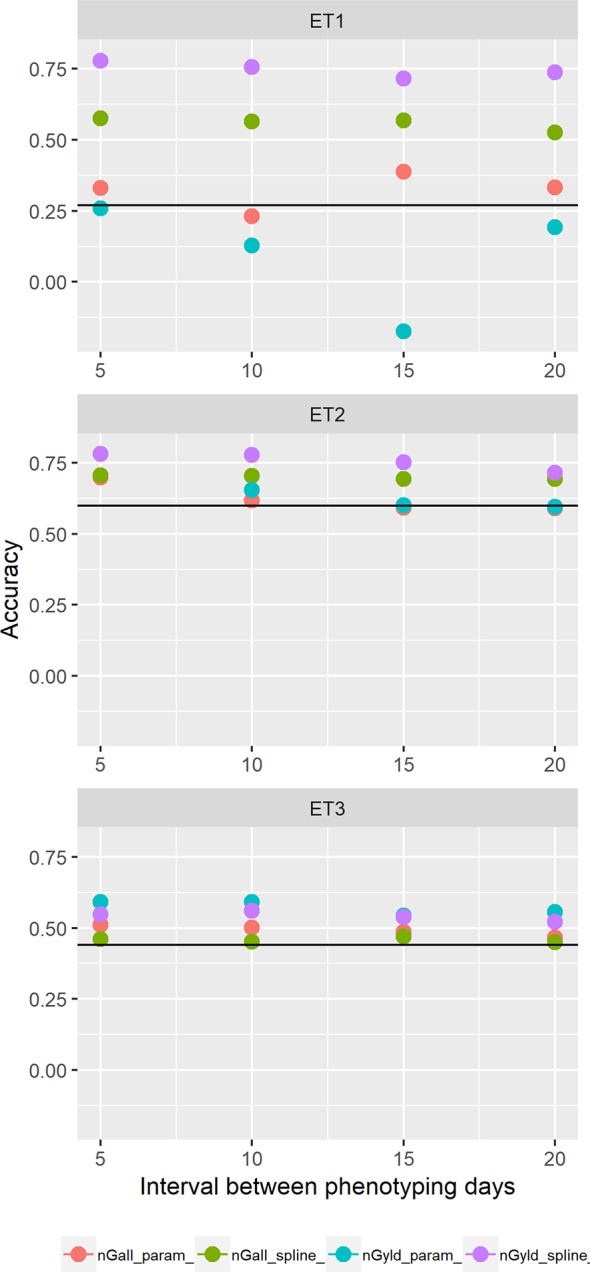
Yield prediction accuracy and standard error in ET1, ET2, and ET3 calculated with the multi-trait prediction models M3S and M3P, considering the target trait and summaries of biomass over time, when*biomass had an error that was a function of canopy cover*. Predictions indicated with nGall_param and nGyld_param considered yield plus BL_asy, BL_slope, and CL_max, and models nGall_spline and nGyld_spline considered yield plus BS_asy, BS_slope and CS_max. The x-axis indicates the interval between consecutive phenotyping days. Symbol color indicates the combination of prediction scenario (*nG_all*, target and secondary traits missing in the validation set or *nG_yld*, target trait missing in the validation set, but secondary traits are present in both training and validation set) and method to model biomass over time (spline or logistic model) interval, expressed as the number of days between two consecutive HTP measurements. Black horizontal lines shows yield prediction accuracy for a single trait model trained with yield data for the genotypes in the training set (M1). Single- and multi-trait models were trained with 100 genotypes, whereas 99 genotypes were used for validation. Bars indicate the confidence interval for the mean, calculated across 30 realizations of the training-validation sets.

We also compared prediction scenarios that differed in the traits that were missing in the validation set: In *nG_all*, yield and secondary traits were missing in the validation set, whereas in *nG_yld*, yield only was missing in the validation set. The increase in prediction accuracy was larger for the scenarios *nG_yld* (**Figure 10**), than for *nG_all* (**Figure 9**), particularly in ET3, probably because green canopy cover in this environment had more genotypic variation due to the earlier onset of senescence under drought. The heterogeneity in the measurement error size over time did not have a large impact on prediction accuracy for the target trait. Prediction accuracy was similar for the phenotyping schedules with a homogeneous error size (**Figure 9**) and for schedules that had a measurement error depending on green canopy cover (**Figure 11**).

### Multi-Trait Predictions Using Physiological APSIM Parameters

Besides the parameters of biomass and green canopy cover over time, we also included APSIM parameters in the multi-trait prediction model; i.e. y_rue (radiation use efficiency), photop_sens (sensitivity to photoperiod) and vern_sens (vernalization requirements). We added a range of error sizes to these parameters to evaluate its impact on prediction accuracy.

**Figure 12:**
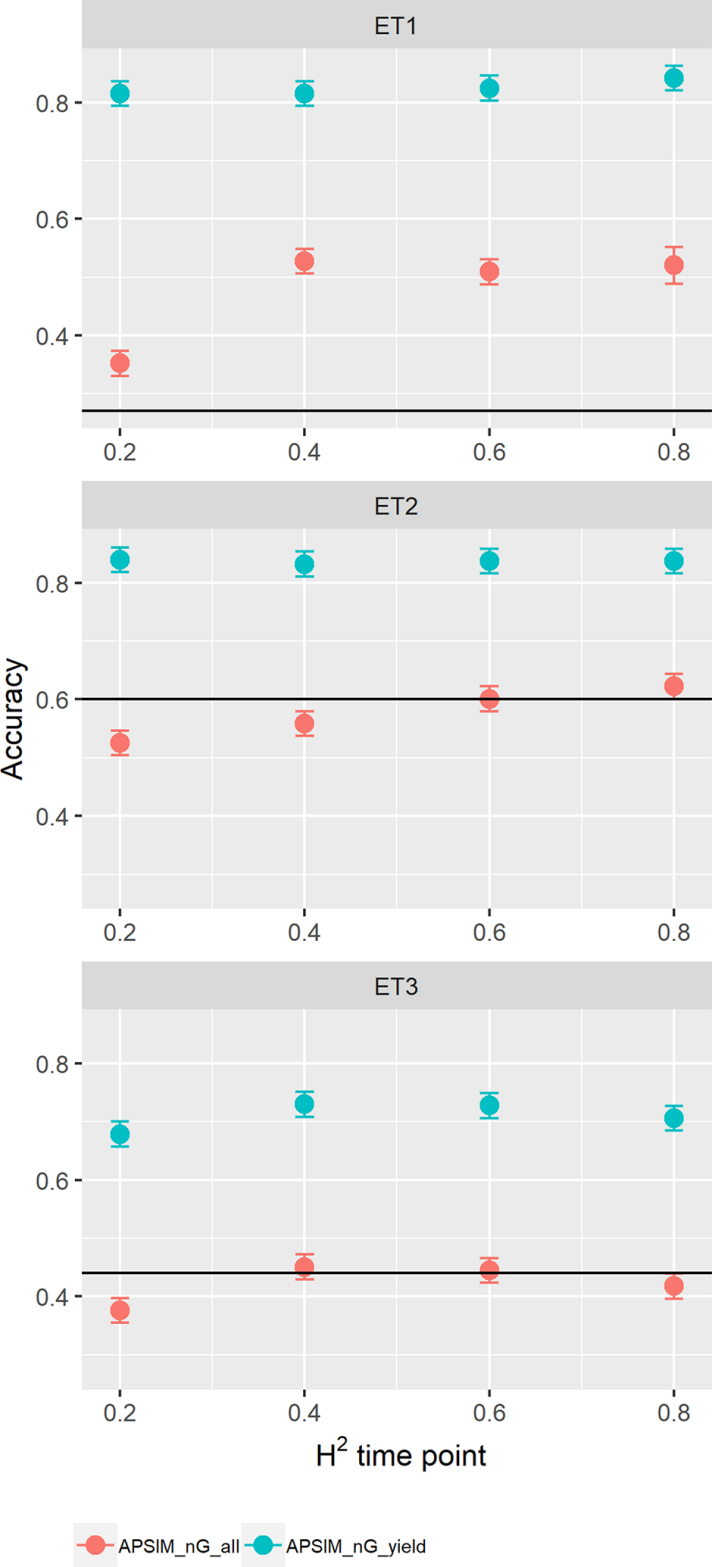
Yield prediction accuracy and standard error in ET1, ET2, and ET3 calculated with a multi-trait prediction model (M4) considering either the target trait and three APSIM (y_rue, photo_sens, and vern_sens). The x-axis indicates the heritability of the HTP measurement for the APSIM parameter. Symbol colour indicates the prediction scenario; *nG_all* (target and secondary traits missing in the validation set) or *nG_yld* (target trait missing in the validation set, but secondary traits are present in both training and validation set). Black horizontal lines shows yield prediction accuracy for a single trait model trained with yield data for the genotypes in the training set (M1). Single- and multi-trait models were trained with 100 genotypes, whereas 99 genotypes were used for validation. Bars indicate the confidence interval for the mean, calculated across 30 realizations of the training-validation sets.

When assessing the effect of including APSIM physiological parameters in the multi-trait prediction model, prediction accuracy increased (**Figure 12**). The increase was observed only in the scenario *nG_yld*, where prediction accuracy reached 0.70 in ET3 and 0.85 in ET1 and ET2. The increase was more modest for the scenario *nG_all*, showing that including basic traits is particularly useful for unobserved genotypes. The advantage of including basic traits is that, as they correspond to response mechanisms to the environment, they tend to have less G × E. Therefore, they need to be phenotyped in a reduced number of environments, compared to secondary traits, and can potentially be useful across a larger number of environments (they are less environment-type dependent than the secondary traits that have a larger amount of G × E).

## Discussion

In this paper, we illustrate how the output APSIM crop growth model with simulated genotype-dependent physiological parameters allows to evaluate the potential of secondary traits measured with HTP to improve yield genomic prediction accuracy. Our APSIM simulations produced daily output for several traits in three environments that were contrasting in their water deficit patterns. We added error to this output to simulate biomass and canopy cover in a range of phenotyping schedules. Then, we used statistical models (parametric and P-splines) to model biomass and canopy cover over time. We estimated parameters that summarize the dynamics of biomass and canopy cover and used these parameters (together with yield data) to evaluate multi-trait prediction models. In our illustration, we used a diversity panel that represents well the spectrum of genotypes that is adapted to Australian environments. However, our approach could also be applied to other panels or population types. We based ourselves on previous research done by Casadebaig et al. (2016), Chenu et al. 2011; 2013, and Zheng et al. (2013)to select a number of environments that represent well the Australian TPE. Similar approaches to characterize yield responses across environments and to identify traits that are useful for selection in different environment types have been previously discussed by (Podlich and Cooper, 1998; Chapman et al., 2002; Hammer et al., 2002; Hammer et al., 2005; Chapman, 2008).

### Multi-Trait Predictions

Yield prediction accuracy was better for multi-trait than for single-trait models. The multi-trait models considered parameters for the dynamics of biomass and green canopy cover as a correlated traits, or APSIM physiological parameters. The only cases in which multi-trait prediction did not have larger accuracy than single trait prediction were for those cases in which the measurement error was very large, or when the curve (in particular the logistic curves) did not fit well to the seasonal trend in biomass data. The degree of success of our predictions was largely affected by the environmental conditions; i.e. biomass- and canopy-related traits are more helpful to increase yield prediction accuracy in non-dry environments, than in dry environments. These results coincide with experimental data showing that normalized difference vegetation index (NDVI) was more beneficial for yield prediction accuracy in non-dry than in dry environments (Rutkoski et al., 2016). The observed trait correlations agree with the experimental observations of Hassan et al. (2019), who showed that correlations between biomass-related traits decrease under drought. The fact that the correlations were stronger for the maximum biomass accumulation rate (BS_slope and BL_slope) than for final biomass (BS_asy and BL_asy) is probably related to the influence of biomass around flowering on the number of grains set (González et al., 2011). Because environmental conditions modify the correlations between the secondary and target traits, the phenotyping and prediction strategy needs to consider the type of environments in which genotypes are evaluated. Our modeling approach allows estimating a priori whether the traits are likely to be correlated with the target trait, and to evaluate trait correlations over a large sample of the TPE, allowing to assess the potential of intermediate traits for making predictions. As an illustration, we focused on a selection of three environments, but our modeling approach could be applied to large data sets across the whole TPE, as shown in Bustos-Korts et al. (2019).

Besides the constant size of the measurement error over time, we also assessed a measurement error dependent on canopy cover, as most commonly encountered in real experiments (Grieder et al., 2015; Magney et al., 2016). We used parameters extracted from P-splines logistic curves fitted to biomass and green canopy cover as correlated traits. Multi-trait prediction models using parameters obtained from biomass with an error that changes as a function of green canopy cover showed similar results, compared to the curve parameters obtained using a constant error size. This result shows the potential of our approach to also evaluate phenotyping techniques that have heterogeneous measurement error. We assumed that errors were uncorrelated over time. If there are reasons to assume that the error, besides changing in size, is also correlated over time, this could be taken into account using an autoregressive or an ante-dependence model for the error (Zimmerman and Núñez-Antón, 2009; Funatogawa and Funatogawa, 2019; Giri et al., 2019).

### Simultaneous Modeling of Traits Measured With HTP During the Growing Season

We modeled biomass as measured with HTP during the growing season. We compared the use of parametric models (logistic functions and cubic model) and P-splines to characterize biomass dynamics over time. Both, P-splines and parametric models, increased the heritability of biomass, indicating that modeling multiple time points simultaneously is a good strategy to reduce the measurement error. Similar results have been observed when using P-splines to model canopy temperature and NDVI measurements in real wheat experiments (Sun et al., 2017). We observed that parameters estimated from P-spline fits have a larger heritability than parameters obtained from logistic curve fits because they can accommodate better the irregularities in the biomass accumulation.

The advantage of using simulated data is that we can evaluate error sizes and measurement frequencies, allowing to provide recommendations for a phenotyping schedule. In our simulations, we covered a range of phenotyping scenarios, varying in their measurement precision and interval, and on the genotypes that are phenotyped (i.e. *nG_all* and *nG_yld* scenarios). For both scenarios, *nG_all* and *nG_yld*, the prediction accuracy for multi-trait models using biomass information during the whole growing season was in line with results obtained for real phenotypic data. For example, Sun et al. (2017)show that canopy temperature and NDVI can be useful to improve yield prediction of genotypes that do not have observations for any trait (*nG_all*), whereas Crain et al. (2018)also observed an increase in yield prediction accuracy when NDVI and canopy temperature were measured in genotypes in the validation set.

Some of the levels we chose for measurement error are perhaps too optimistic, given that biomass approximations with technologies like NDVI usually have R^2^ of maximum 0.60 (Marti et al., 2007; Grieder et al., 2015; Magney et al., 2016). Our results indicated that when integrating the information over the growing season, similar prediction accuracy is obtained when using HTP technologies that deliver an R^2^ of 0.60 or 0.80. This suggests that, if we use the currently available technologies, more can be gained from the integration of multiple observation during the growing season, than from reducing the error of single observations. The next step in terms of integration of HTP data into phenotype prediction might be combining the information from proximal sensing of field trials (e.g. NDVI measured from a drone or helicopter, Chapman et al., 2014) with remote sensing from the actual wheat production environments (e.g. satellite measurements of wheat paddocks, Perry et al., 2014).

### Trait Hierarchy, Physiological Breeding, and Multi-Trait Prediction

Intermediate traits, in general, express a larger G × E, compared to basic traits (Bustos-Korts et al., 2016b; Bustos-Korts et al., 2019; van Eeuwijk et al., 2019). The notion of differences in scale (basic traits with short phenotypic distance to the genetic basis vs. intermediate traits with larger phenotypic distance to the genetic basis) is useful to organize the phenotyping and the breeding strategy (Hammer et al., 2016; Hammer et al., 2019). Secondary traits that have a short phenotypic distance to the target trait (more genetically correlated) are more useful selection targets and they also have a larger potential to be used in multi-trait genomic prediction models. Examples of the use of platforms/controlled conditions to characterize basic traits are wheat early vigour measured in the greenhouse (Duan et al., 2016), the root angle in maize and sorghum measured in greenhouse pots as a trait related to water uptake (Singh et al., 2010), or the sensitivity to photoperiod, vernalization and earliness per se in wheat measured in controlled conditions for photoperiod and temperature (Zheng et al., 2013; Sukumaran et al., 2016). Examples for intermediate traits in field conditions are airborne measurements for wheat NDVI and canopy temperature (Deery et al., 2016; Rutkoski et al., 2016). These traits can be combined by pyramiding their underlying alleles, in a strategy called “physiological breeding” (Reynolds et al., 2009). To facilitate this process, we propose the convenience of using platforms, greenhouses or facilities with more controlled conditions for detailed phenotyping of basic traits, and field phenotyping for the more integrative traits, that commonly show a larger G × E. These basic traits could then be used in a prediction scenario like *nG_yld*, helping to increase yield prediction accuracy across environments. Scenario *nG_yld* would also be analogous to the idea of “phenomic selection” proposed by Rincent et al. (2018), in which the phenotypic data is used as a proxy of the SNP data to estimate the genotypic similarity between individuals.

Data from field imaging for integrative traits and from platforms for basic traits (i.e. crop growth models parameters) can be used for predicting target traits. Different approaches are possible. The first type of phenotyping network would rely on a central location to intensively phenotype basic traits in platforms, with some additional phenotyping of integrative traits in the field. As basic traits are commonly difficult to measure, phenotyping could be made on a few genotypes, using genomic prediction to predicting the rest of the target population of genotypes (Pauli et al., 2016). This strategy is also highly attractive for genomic prediction, where the expensive basic trait is measured on a subset of genotypes that represent the relevant genetic space of the target population of genotypes (Albrecht et al., 2014; Bustos-Korts et al., 2016a) and then the rest of the population can be predicted from a training set. Under this scheme, prediction of the target trait would require a good articulation of statistical and crop growth models. Another application would be the use of secondary traits to improve prediction accuracy across breeding cycles. For example, Sun et al. (2019) use canopy temperature and NDVI measured in early breeding stages to improve yield prediction accuracy in later stages. We propose that our simulation methodology could be used to evaluate a large number of prediction scenarios, considering a large range of trait and measurement error combinations, narrowing down the range of phenotyping and prediction scenarios that need to be evaluated empirically during the design process of the phenotyping protocol.

### Environment Classification

We examined the genetic correlations between yield, parameters for the biomass and canopy dynamics, and with the APSIM physiological parameters. We showed that genetic correlation between traits is time- and environment-dependent. In this paper, we focused on three environments only, but the same approach could be considered across the whole TPE to study the consistency of the correlation patterns across environments. For example, Bustos-Korts et al. (2019)show that the correlation between yield and the underlying traits biomass and phenology changes over time. However, the temporal pattern is very similar for environments that have similar environmental conditions. The time- and environment dependencies of trait correlations inform about the physiological adaptation mechanisms that are relevant to each of the environment types. Therefore, trait correlations could also be used as a diagnostic tool to classify environments, assuming that environments with similar environmental conditions will induce similar trait correlations. To answer this question, techniques like clustering methods or networks could be applied on phenotypic data of multiple traits.

## Conclusions

The combined use of crop growth models and multi-trait genomic prediction models provides a procedure to assess the efficiency of phenotyping strategies and to evaluate the impact of yield components under different environment types on the genomic prediction of final yield.

Using P-splines or parametric models to extract parameters that characterize the dynamics of secondary traits allows to increase trait heritability, compared to individual time points. This increases the potential of secondary traits to achieve a larger prediction accuracy for the target trait.

Yield prediction accuracy benefitted from including biomass and green canopy cover parameters in prediction scenarios with no- or limited water stress. The advantage of the multi-trait model was smaller for the early-drought scenario, due to the reduced correlation between the secondary and the target traits.

## Data Availability Statement

The simulation output can be accessed by request.

## Author Contributions

DB-K defined the simulation settings, run the simulations, did the modeling of the simulation output, and wrote the first manuscript draft. MB did the modeling of traits over time and wrote parts of the manuscript. MM defined the simulation settings and prediction models. SC provoked the original ideas for this work and defined the simulation settings. KC defined the simulation settings and provided the APSIM simulation files. BZ provided input about the error ranges. FE provoked the ideas, helped to define the simulation settings and prediction models, provided ideas for the general modeling framework, and wrote part of the manuscript.

## Funding

This paper received financial support of the European Union's Seventh Framework Programme (FP7/ 2007-2013) under the grant agreement n°FP7-613556, WHEALBI and from the EU project H2020 817970 (INVITE). DB-K received financial support from Becas Chile (Comisión Nacional de Investigación Científica y Tecnológica, CONICYT, Chile).

## Conflict of Interest

The authors declare that the research was conducted in the absence of any commercial or financial relationships that could be construed as a potential conflict of interest.
